# Challenges in conducting psychiatry studies in India

**DOI:** 10.4103/2229-3485.76284

**Published:** 2011

**Authors:** Saifuddin Kharawala, Jeroze Dalal

**Affiliations:** *Clinical Research Consultant, Consultant Psychiatrist, GlaxoSmithKline Pharmaceuticals India Ltd., Mumbai, Maharashtra, India*; 1 *Clinical Operations, GlaxoSmithKline Pharmaceuticals India Ltd., Mumbai, Maharashtra, India*

**Keywords:** Challenges, India, informed consent, placebo, psychiatry, rating

## Abstract

A large number of psychiatry studies are conducted in India. Psychiatry studies are complex and present unique challenges in the Indian setting. Ethical issues pertaining to the risk of worsening of illness, use of placebo and validity of informed consents are commonly faced. Site selection can be difficult due to the relative paucity of ICH-GCP (International Conference on Harmonisation - Good Clinical Practice) trained psychiatry investigators in India. Recruitment can be challenging due to issues such as strict eligibility criteria, (lack of) availability of caregiver, illness-related considerations, etc. Assessment of the consent capacity of patients is not simple, while structured assessments are not commonly employed. As the illness fluctuates, the consent capacity may change, thus requiring continued assessment of consent capacity. Study patients run the risk of worsening of illness and suicide due to exposure to inactive treatments; this risk is counterbalanced by use of appropriate study designs, as well as the indirect psychotherapeutic support received. Psychiatry studies are associated with a high placebo response. This necessitates conduct of placebo-controlled studies despite the attendant difficulties. Also, the high placebo response is often the cause of failed trials. Rating scales are essential for assessment of drug response. Some rating instruments as well as some rater training procedures may not be suitable for the Indian setting. Technological advancements may increase the procedural complexity but improve the quality of ratings. Psychiatry studies present monitors and auditors with unique scenarios too. Utilization of psychiatry specific training and expertise is recommended to ensure successful conduct of these studies in India.

## INTRODUCTION

Nearly one out of three Indian studies listed on the United States Federal Drug Agency (USFDA) website has been conducted in psychiatric indications (Behaviors and Mental Disorders).[1] The large pool of genetically diverse, often treatment-naïve patients has contributed to India being a favored site for psychiatry studies.

Psychiatry is a complex subject and this complexity extends into research and development too. There are challenges involved from the point of identifying new chemical entities and conducting preclinical and clinical studies to analyzing the data from trials and understanding their applicability to patient care.[2] In this article, we shall focus primarily on the operational challenges that are faced in conducting psychiatry clinical studies in India, with an emphasis on those issues encountered during industry sponsored multicentric studies.

## ETHICAL ISSUES

Mentally ill patients are a vulnerable group because mental illness impacts the decision-making capacity of the sufferers. In addition, these patients are often subject to much stigma owing to the illness.[3] The subject of psychiatry is complex and lends itself to many ethical dilemmas.[4] Ethical concerns primarily center on the following issues – risks that the patients are exposed to during clinical studies, the rationale of conducting placebo-controlled studies, and the validity of the patients’ consents.[5–7] As a result, psychiatry studies conducted in developing countries such as India attract considerable scrutiny.[8]

It should be noted that more than half the total US FDA inspections in India have been conducted in Neuropsychiatry – this includes nine consecutive inspections from April 2007 to September 2009.[9]

## INVESTIGATOR AND SITE SELECTION

Although a large number of psychiatry studies are conducted in India, the pool of investigators is quite limited. There are only about 4,000 psychiatrists for 6.5 crore mentally ill patients in India.[10] This number is inadequate both for routine clinical management as well as for clinical research. Of the total psychiatrists, not more than 100 are ICH-GCP (International Conference on Harmonisation - Good Clinical Practice) trained with experience in conducting trials. As a result, the few high-quality experienced sites are often saturated with studies; it is not uncommon for the most experienced sites to, on occasion, be conducting over 10 simultaneous studies with many of them being in the recruitment phase. This can be particularly challenging as psychiatry studies are in general more complex and demand more time than studies in most other therapy areas.

Site selection is also limited by other factors. We will be discussing later in the article how some psychiatry study designs mandate the use of independent raters for compelling methodological reasons. Raters in psychiatry are usually psychiatrists or psychologists, and it is often difficult for sites to arrange for such independent raters in addition to the regular site staff that are required for conducting the studies.

## CHALLENGES WITH RECRUITMENT

### Eligibility criteria

In general, exclusion criteria in psychiatry studies tend to be stricter than involving studies in other therapy areas. Exclusion criteria that are unique to psychiatry include exclusion of patients with substance abuse, certain personality disorders, those at risk of suicide, etc. These can lead to up to 90% of patients being excluded on the basis of the eligibility criteria alone.[11]

### Lack of documentation

There are no diagnostic laboratory tests for mental illnesses. The diagnosis primarily rests on the history obtained from the patient and caregivers, and the mental state examination (MSE). However, international trials often require documented evidence of previous episodes as an eligibility criterion. This can be quite challenging as many patients in India have nothing more than their medication strips to show for their previous episodes and treatments.

### Role of caregiver

In India, most mentally ill patients are accompanied by a caregiver when they come for treatment. In clinical studies, patient visits are more frequent and of longer duration than in general clinical practice. This puts an added strain on the caregivers who may be earning members and may already be struggling to adjust with the reduced productivity resulting from the illness in the family. They find it difficult to spend the extra time mandated by the study participation and this precludes the patient’s participation in clinical studies.

### Screen failures

Failed drug screen tests are much more common in psychiatry than in other therapy areas. In India, patients tend to visit psychiatrists only after having already received treatment from general practitioners or faith healers. Such patients may have been prescribed psychoactive substances like benzodiazepines. This can alter their mental health status and would show on the drug screen tests, thus leading to screen failures. Also, substance abuse and substance dependence are common co-morbidities with psychiatric illnesses and it is not very uncommon to find the drug screen test being positive for cannabis or benzodiazepine as a result of substance abuse.

### Illness factors

The illness itself may impact recruitment in some cases.[4] In paranoid schizophrenia, patients are suspicious and are inclined to view any experimental treatment with mistrust. Non-motivated depressed patients with psychomotor retardation may find it difficult to decide whether to participate in a study or not. Similarly, patients with obsessive–compulsive symptoms may be caught in ambivalence and may be unable to make a decision regarding study participation.

## INFORMED CONSENT

The informed consent process is probably the most controversial aspect of conducting psychiatry studies. For a satisfactory informed consent procedure, the patient needs to be of sound mind and should be able to both understand the information presented as well as make a sound judgment. However, psychiatric illnesses affect multiple aspects of the mental functions like mood, attention, concept formation, etc. all of which impact the “consent capacity” of patients.[12]

We may consider some examples to better understand this issue. A patient suffering from psychosis may lose touch with reality and therefore not realize that he is suffering from an illness; he may thus see no need for any treatment at all, leave alone treatment in a clinical trial. A patient suffering from mania may have impaired judgment and may be predisposed to take undue risks; this could adversely influence his assessment of the risks involved in participating in a trial. In depression, cognitive functions such as attention and information processing are impaired, thus making lengthy complex legally worded informed consent documents more difficult to comprehend.

The examples given above are simplifications because some impairments in cognition, judgment, insight, etc. are seen in nearly all psychiatric illnesses. Also, the severity of these impairments varies from patient to patient, and from time to time. The investigator is then required to make a judgment regarding “consent capacity” on a case by case basis. This is a challenging task and involves the participation of the patient’s legally acceptable representative (LAR) on those occasions when the investigator deems the patient unfit to make an informed decision.[6]

Another aspect regarding informed consent which bears scrutiny is continued consent.[13] In general, informed consent is taken initially and then repeated whenever there are amendments to the consent document. In psychiatry, however, continued consent has an important role. Patients who possessed consent capacity at the beginning of the study may lose this capacity if their illness worsens during the course of treatment. Such patients could, for example, lose the capacity to choose to withdraw from the study at any time. The investigator needs to be alert to this possibility and needs to monitor the consent capacity of the patients on an ongoing basis. Where indicated in such situations, he may transfer the consent and decision-making duties to the LAR.[14] The reverse may also be true. A patient who did not have consent capacity at the start of the study may show improvement during the course of treatment and reach a stage where he is able to make an informed decision for himself. In such a case, the decision-making duties should be transferred from the LAR to the patient in a timely manner.

In view of the complexities involved, some authorities recommend a more detailed and structured assessment of the consent capacity of mentally ill patients participating in research.^14^,^15^ In the absence of such strategies, the consent obtained in psychiatry studies will continue to remain open to questioning and doubt.[7,8]

## RISK OF WORSENING OF ILLNESS AND SUICIDE

Another challenging aspect of conducting clinical studies in psychiatry is dealing with the risk of worsening of illness and suicide.[5] During a clinical study, mentally ill patients are exposed to inactive (placebo) substances and experimental compounds. This may lead to worsening of the underlying illness which could potentially precipitate suicidal behavior. Also, many psychiatry protocols include a washout period. This is usually done to eliminate lingering effects of previous psychoactive medications prior to baseline assessments. The washout period can be potentially dangerous as the patient does not receive any treatment during this period except occasional rescue medications when indicated. Hence, psychiatry studies, particularly those dealing with illnesses such as depression, need to have in place elaborate measures in the protocol to ensure that the risk of suicide is minimized.

Despite the perceived risks, however, the current available data do not suggest any increased risk of suicide in patients receiving placebo in depression studies.[16,17] In fact, the risk of suicidal behavior during clinical studies is much lesser than that seen in real life. One obvious reason for this is that protocols carefully exclude patients with risk factors for suicidal behavior. In addition to this, the patients also receive indirect psychotherapeutic support because of the increased frequency and duration of visits during clinical studies, and this has a protective effect.

## PLACEBO RESPONSE

One of the most challenging and difficult to manage aspects of psychiatry studies is controlling the placebo response. Simply put, placebo response is the response or improvement seen in patients who are on the placebo arm of the trial. Placebo response occurs due to various reasons. One reason is the indirect psychotherapeutic support mentioned earlier. Another reason is the patient’s and rater’s expectation of improvement during the course of treatment. Statistically, some placebo response occurs as a result of regression to the mean. Finally, some patients may experience spontaneous improvement in symptoms.

In psychiatry studies, the placebo effect is of significant proportion and is increasing over time. A recent meta-analysis found that the placebo response in depression studies ranged from 10 to 50% with an average of 30%[18] This implies that an experimental treatment may show a response rate as high as 50% even if it was ineffective. As a result, it becomes necessary to conduct placebo-controlled studies. Merely demonstrating response is not sufficient; only if a drug is significantly better than placebo can it be considered effective.

Concern about placebo induced worsening of symptoms has led to suggestions that any new psychoactive medication should be studied against an active comparator only. However, studies conducted without the placebo group would involve much larger numbers, thus exposing many more patients to potential risks. Thus, for ethical and methodological reasons, placebo-controlled studies are essential, and the key is to manage the potential risks appropriately.

High placebo response is one of the main causes of failed late phase trials.[19] The lesser the placebo response, the better the possibility of accurately knowing if the experimental drug is effective or not. The challenge then is to limit the placebo response. From the operational point of view, one of the most important aspects that impact placebo response is the administration of rating scales.

### Rating scales – Subjective to objective

The absence of “hard” diagnostic criteria in psychiatry creates unique challenges. Diagnosis is made on the basis of diagnostic criteria such as the Diagnostic and Statistical Manual (DSM) criteria. Assessment of improvement primarily relies on the use of rating scales, for example, the Montgomery–Asberg Depression Rating Scale (MADRS) for depression. These rating scales help to convert the subjective improvement into objective data which can then be analyzed and assessed.

The challenge is to ensure that the rating scales capture only the actual change in the illness due to the active drugs and not the non-specific placebo effects. To this end, raters in psychiatry studies undergo rigorous training including didactic training, rating of recorded interviews and conduct of supervised interviews. However, the “patients” in such trainings (whether real patients or actors) are usually western patients. As a result, the lessons that Indian raters learn from such trainings are not completely culturally applicable to the clinical settings in India. Similarly, psychiatry rating instruments and structured interview guides are usually developed in keeping with the western culture, and some of them may not be culturally appropriate for non-western patients even after translations into local languages.[20] This, besides making the conduct of the studies more difficult, also adversely impacts the validity of the instruments, and therefore, the quality of data obtained from Indian subjects.

Recent innovations in this area include the use of independent raters, cross-over raters, use of Interactive Voice Response System (IVRS), video conferencing with centralized raters, audio taping of interviews for quality checks, etc.[21–24] Newer and possibly more complex techniques to improve data quality are likely to be introduced if studies continue to fail due to high placebo responses. All these innovations are expected to bring forth new technological challenges for the conduct of psychiatry studies with unique implications for sites in India.

## MONITORING AND AUDITING

As a result of the complexities outlined above, psychiatry studies are often a challenge to monitor and audit. Assessment of inclusion and exclusion criteria from the history and MSE is not simple. The MSE is unique to psychiatry; here the monitor/auditor is exposed to many new terms with very specific meanings. In psychiatry studies, the monitor/auditor would need to address various subtle questions such as – “Is there adequate documentation of the patient’’s judgment capacity?”, “For patients who are deemed to have ’poor insight’ or ’impaired judgment’, is there adequate documentation demonstrating the impact of these features on the informed consent process”, “Was there any risk of suicidality at any time?”, “Is there any contradiction in the patient’s MSE conducted by the investigator and the independent rater?”, “Is the investigator judging the continuation of consent throughout the study?”, “Are drop-outs being followed up intensively in view of the potential risk of suicide?”, etc.

Thus, monitors and auditors face issues that are very different from those in other therapy areas, and it is a challenge to master the art of decoding and understanding psychiatry.

In conclusion, conducting psychiatry studies in India can be quite a challenging task. The field of mental health itself is complex, and the milieu in India presents unique challenges. Lessons learnt from experience with other therapy area studies are not always directly transferable to psychiatry studies. Additional psychiatry-specific training and expertise is often required. A perceptive and deep understanding of patient vulnerabilities, ethical issues, and methodological difficulties is essential for the successful conduct of clinical studies in psychiatry.
